# Tuberculosis and Autoimmunity

**DOI:** 10.3390/pathophysiology29020022

**Published:** 2022-06-13

**Authors:** Irina V. Belyaeva, Anna N. Kosova, Andrei G. Vasiliev

**Affiliations:** Department of Pathophysiology, St. Petersburg State Pediatric Medical University, Ministry of Healthcare of the Russian Federation, 194100 Saint Petersburg, Russia; annainet@yandex.ru (A.N.K.); avas7@mail.ru (A.G.V.)

**Keywords:** tuberculosis, autoimmunity, cell death, adjuvant, vitamin D, cytokines, genetic polymorphism

## Abstract

Tuberculosis remains a common and dangerous chronic bacterial infection worldwide. It is long-established that pathogenesis of many autoimmune diseases is mainly promoted by inadequate immune responses to bacterial agents, among them Mycobacterium tuberculosis. Tuberculosis is a multifaceted process having many different outcomes and complications. Autoimmunity is one of the processes characteristic of tuberculosis; the presence of autoantibodies was documented by a large amount of evidence. The role of autoantibodies in pathogenesis of tuberculosis is not quite clear and widely disputed. They are regarded as: (1) a result of imbalanced immune response being reactive in nature, (2) a critical part of TB pathogenicity, (3) a beginning of autoimmune disease, (4) a protective mechanism helping to eliminate microbes and infected cells, and (5) playing dual role, pathogenic and protective. There is no single autoimmunity-mechanism development in tuberculosis; different pathways may be suggested. It may be excessive cell death and insufficient clearance of dead cells, impaired autophagy, enhanced activation of macrophages and dendritic cells, environmental influences such as vitamin D insufficiency, and genetic polymorphism, both of Mycobacterium tuberculosis and host.

## 1. Introduction

Tuberculosis (TB), a dangerous chronic infectious disease caused by *Mycobacterium tuberculosis* (Mtb), is still a threat to public health worldwide. A global total of around 10 million people became ill with TB in 2020 [1]. Drug resistance of Mtb [2], HIV infection, malnutrition, especially vitamin D deficiency, aging, autoimmune diseases, and abundant usage of immune suppressants contribute to increased incidence of TB [3].

Epidemiological studies associate microbial infections and autoimmunity (AI), hypothesizing infections to be able to trigger autoimmune diseases (AID) [4,5,6]. A number of studies have shown sera from patients with active TB to contain autoantibodies (AAB). TB has many different outcomes and complications. Autoimmunity (AI) is one of the processes characteristic of TB; at least, the presence of AABs was documented by a large amount of evidence. AABs, being typical for autoimmune disorders, are also present in different infectious diseases [5,6,7,8]. The role of AABs in the pathogenesis of TB development is widely disputed. They are considered (1) as a result of imbalanced immune response being reactive in nature [9,10,11]; (2) as a critical part of TB pathogenicity, leading to cavitation and transmission [12]; (3) as a beginning of AI disease [12,13]; (4) as a protective mechanism helping to dispose of microbes and infected cells [14]; and (5) as playing a dual role, pathogenic and protective [14]. Such diverse opinions lead to the conclusion that mechanisms involved may vary in each case. Mtb can trigger different pathways of the immune responses.

Several possible mechanisms of AI development in TB may be suggested. It may be excessive cell death and insufficient clearance of dead cells, impaired autophagy, enhanced activation of macrophages (Mphs) and dendritic cells (DCs), environmental influences such as vitamin D insufficiency, and genetic polymorphism, both of Mtb and host. Chronic presence of infection can be regarded as an endogenous adjuvant [15]. With the existence of different pathways of immune responses, the one receiving the support from additional factors dominates. Multiple surface Mtb molecules can differently orchestrate immune responses.

Little is known about mechanisms of autoimmunity development in TB; the knowledge is mainly “Lessons Learned from Autoimmune Diseases” [16].

The unique mechanism of AAB generation involving the autoreactive B-cells expressing T-bet transcription factor has been identified for classic AIDs and microbial infections [17,18,19]. The recognition of a nucleic acid by toll-like receptor 7 (TLR7) and synergistic stimulation by IFNγ of B cells lead to the induction of T-bet+ B-cells and production of IgG2a [20]. T-box transcription factor T-bet being protective against intracellular pathogens is prone to producing AABs [18].

Antiphospholipid antibodies (aPL) were detected in different AIDs and infections such as TB (reviewed in [21,22]). Lipid molecules stimulate innate-like B-1 B cells to antibody production [23]. They react with self-determinants, such as carbohydrates and glycolipids, and often cross-react with bacterial antigens. Phospholipids are major antigens stimulating B-1 B cells [23]. The IgM production by B-1 B cells requires long-term stimulation by lipid antigens of replicating mycobacteria [24]. 

Mycobacterial lipids have been shown to act as adjuvants. Complete Freund’s adjuvant (CFA), which includes components of Mtb and has a high adjuvant activity, is used in mice for the induction of AIDs such as experimental autoimmune encephalomyelitis (EAE) and uveitis [25]. Lipid components have been found to be essential for CFA’s adjuvant activity [26].

Mtb is recognized by multiple phagocytic receptors, among them pattern-recognition receptors, especially the TLR on Mphs and DCs. Polymorphisms in TLRs affect human susceptibility to TB [27,28] and may be associated with AI.

The genome of Mtb has been shown to encode a protein family PE/PPE/PGRS, present exclusively in the genus Mycobacterium [29]. The PE/PPE/PGRS proteins influence cell-envelope remodeling, host cell-death pathways and virulence [30], mycobacterial antigenic variation, immune evasion [31], innate immunity, and bacillary survival in Mphs [32,33]. Polymorphisms in the PE/PPE/PGRS protein family may influence different manifestations of TB, among them AI. 

Cell death is an essential physiological and pathological process influencing the coordination of immune responses and AI [34]. Apoptosis of infected cells results in self-reactive T-cell promotion of AI in infections [35], and excessive Mph apoptosis in TB may potentially cause a most important mechanism. Mer tyrosine kinase (MerTK) has been reported to be a major Mph apoptotic-cell receptor, its functional defect causing inadequate AC clearance promoting AI and atherosclerosis [36].

Phagocytosis of infected apoptotic cells has recently been shown to result in simultaneous presence of both cellular and microbial antigens inside the same phagosome. This makes possible the presentation of self-antigens by MHC II molecules, causing generation of autoreactive Th17 cells, associated with AAB production [35]. 

Pyroptosis, manifesting by osmotic lysis and releasing distracted remnants and inflammatory cytokines [37], is characteristic of TB [38]. Pyroptotic cells release an important inflammatory protein high-mobility group box 1 (HMGB1) [39]. Complex HMGB1- DNA in vitro contributes to autoreactive B-cell formation [40]. Cytokines caspase 1-dependent IL-1b and IL-18 released by pyroptotic cells are thought to play a role in promoting AIDs [41]. 

Mycobacteria are known to modulate the host cell’s death. Apoptosis, pyroptosis, autophagy, and necrosis were documented in TB [42]. Many of the PE/PPE/PGRS family proteins of Mtb affect these types of cell death in TB [29,43,44,45].

Infections can be connected with the onset of SLE(systemic lupus erythematosus) [4,5,37]. Clearance deficiency may link infections with AIDs [4], SLE [46], and ANCA (Antineutrophil cytoplasmic antibody)-associated vasculitis [47].

High titers of various AABs are present in pulmonary TB patients with vitamin D deficiency [48,49,50,51]. Vitamin D deficiency was registered in multiple sclerosis (MS) [52,53], rheumatoid arthritis (RA) [54,55,56,57], and inflammatory bowel disease [53,57,58,59]. The role of vitamin D in autoimmune diseases was demonstrated in [57,60]. Vitamin D status and polymorphisms of vitamin D receptor were shown to influence the AID development trend [61].

Several cytokines are associated with AIDs. TGFβ and IL-6 promote the early stage of Th17 cell differentiation in mice [62], while IL-23 is necessary for the functional maturation and maintenance of highly pathogenic Th17 cells [63,64,65] essential for the development of AI [66,67,68]. Subset Th17.1, which is characterized by high pathogenicity in the pathogenesis of AIDs, was also detected in TB patients [3].

Genetic as well as nongenetic factors of both the bacterium and the host may have influence on the host response to *Mycobacterium tuberculosis* [69].

## 2. Occurrence of AABs in Active TB Patient Sera

Early reports have established links between Mtb and AI [7,8,70,71]. A number of studies connecting TB with AI investigated the AAB characteristics of AIDs. The list of AABs includes rheumatoid factor (RF), antinuclear antibodies (ANA), anti-dsDNA AAB, anticardiolipin antibody (ACA; IgM isotype predominant), antineutrophil cytoplasmic antibodies (ANCA), and anticyclic citrullinated peptide (anti-CCP) [8,9,11,48,50,72,73,74,75,76,77,78]. (Table 1) 

Several reports demonstrated the presence in the active TB patients’ antinuclear antibodies [7,8,50,70,72,73], AAB to double-stranded DNA (dsDNA) [10,48,50,77], which are characteristic of SLE. ANCAs, also typical for autoimmune diseases, were revealed in TB patients by different methods and results did not depend on the stage of disease, category of tuberculosis, concomitant diseases, or drug therapy [74]. Another study established an increase in ANCAs and bactericidal/permeability increasing protein in sera of patients with pulmonary TB after treatment [75]. Anticyclic citrullinated proteins and rheumatoid factor were found in patients with active TB [76]. In our investigations [48] the increased level of AABs in TB patients most often occurred with respect to the dsDNA. The TB patients also demonstrated enhanced levels of AABs to different antigens, but AABs to TSH-receptor, to kidney antigens, and to insulin were prevailing. Sera of TB patients were examined [10] for autoantibodies to Ro antigen, La antigen, centromere protein, double-stranded DNA (dsDNA), topoisomerase I (Scl-70), Smith protein, ribonucleoprotein particle (RNP), histone protein, and histidyl-transfer RNA synthetase (Jo1). Anti-Scl-70, antihistone, and anticardiolipin IgG were the predominate autoantibodies in TB patients.

Some authors concluded that AABs present in TB do not lead to clinical manifestations of AIDs, even if AABs were characteristic of certain diseases [10,48,75]. However, the presence of anti-CCP and RF correlated with long fever [75]. Despite high prevalence of AABs to the thyroid gland and the TSH receptor in TB patients, no changes in concentrations of thyroid hormones and TSH were discovered, but a wider range of AABs was found in more severe fibrous cavernous TB than in infiltrative TB [48]. The authors, who demonstrated the presence of AABs to different antigens in the TB patients, suggested that AABs are reactive to TB instead of being pathognomonic, and do not need immunosuppressant therapy [10].

There is also a conflicting report. No valid relationship has been found between AAB prevalence and pulmonary tuberculosis in the case of active pulmonary tuberculosis from Uganda, South Africa, Peru, and Bangladesh [78]. However, AI in TB has been opined to be an essential process driving pathology in tuberculosis, causing cavitation and transmission [12]. 

TB patients may develop noninfectious reactive polyarthritis (Poncet’s disease) or TB rheumatism [9]. The rheumatologic manifestations of TB and the occurrence of TB associated with rheumatologic diseases are summarized in [9].

The opposite relationships were also observed, namely that AIDs enhanced risks of TB. TB has been demonstrated to have autoimmune manifestations such as nodular vasculitis [13], Sjögren’s syndrome, SLE, RA, dermatomyositis, and polymyositis [79]. TB risk in RA patients was found to be about four times higher compared with general populations [80].

## 3. The Unique Pathway of B-Cell Activation Causing IgG2a AAB Production

Recently, a similar mechanism of AAB generation for classic AIDs and microbial infections connected with the autoreactive B-cell population expressing the transcription factor T-bet has been identified [17,18,19,20]. T-bet+ B-cells were found to be major producers of AABs [18]. B cells expressing the transcription factor T-bet may take part in a number of protective and pathogenic immune responses [20]. Both in infectious and classical AI, the mechanism of activation of T-bet+ B-cells involves the recognition of a nucleic acid by toll-like receptor 7 (TLR7) and synergistic stimulation of IFNγ receptors on B cells [17,18]. These signals induce T-box transcription factor T-bet and IgG2a switching in B cells [19].

T-bet has been demonstrated to have an important role in the protective immunity against intracellular pathogens and is prone to producing AABs [20]. T-bet+ B cell induction and expansion were revealed in mouse AI models and in patients with autoimmune diseases such as SLE, MS, RA, Crohn’s disease, and Sjögren’s syndrome [18].

## 4. Antiphospholipid Antibodies

Antiphospholipid antibodies (aPL) were revealed in various clinical conditions (AID) and infections such as TB (reviewed in [21,22,24]). The increased levels of ACA in TB patients were found in several studies [8,10,11,50]. Many viral, bacterial, and parasitic infections can induce aPL, mainly ACA, which do not correlate with thrombosis risk and antiphospholipid syndrome [21].

The elevated concentration of antibodies against β2 glycoprotein IgG and ACA IgG normalized after TB treatment was shown in active TB patients [11]. A significant number of patients had high levels of AABs against proteinase 3 (PR3), myeloperoxidase (MPO), bactericidal/permeability-increasing protein (BPI), and lactoferrin. Most antilactoferrin and anti-MPO levels decreased after treatment, while anti-PR3 increased in most patients [75]. Antiphospholipid antibody levels were suggested to use as biomarker TB treatment in noncavitary TB patients due to their high TB-treatment sensitivity [24].

Phospholipids in the Mtb cell envelope are phosphatidylglycerol, phosphatidylinositol, cardiolipin, and its mannoside derivatives, as well as phosphatidylethanolamine [81]. Because some of them can only be found in mycobacteria, they can be potential biomarkers for diagnosis and treatment response [24,82].

## 5. B-1 B Cells Produce IgM Antiphospholipid Antibodies, Which Have Auto- and Polyreactive Properties

Lipid molecules cause antibody response by B-1 B cells, representing about 5% of B cell population. B-1 B cells express high levels of IgM and do not need T cells for proliferation [23]. B and T cells with self-reactive antigen receptors are usually deleted during their development in order to avoid AIDs. On the contrary, innate-like B-1 cells in mice are positively selected for self-reactivity as long-lived, self-renewing B cells that generate most of the circulating natural IgM [83]. They respond to self-determinants, such as carbohydrates and glycolipids, and often cross-react with bacterial antigens. Major stimulating B-1 B cells antigens are phospholipids [23]. IgM aPL antibodies have self- and polyreactive properties [83]. 

The IgM antibody production by B-1 B cells needs long-term stimulation by lipid antigens generated by replicating mycobacteria during TB. Dead host cells and Mtb cells release enough antigens to activate the B-1 B cells and induce IgM aPL antibody production [24].

## 6. Mycobacterial Lipids Act as Adjuvants

Mycobacterial lipids have been shown to act as adjuvants. Adjuvants are a component in the vaccine stimulating innate immunity and memory-type immunity [25,84]; they are used to establish preferable types of immune responses [84].

Jules Freund created a powerful adjuvant composed of water-in-mineral oil emulsion and heat-killed mycobacteria. CFA, being highly effective, often causes granulomas, sterile abscesses, and ulcerative necrosis at the injection site and cannot be used for humans. CFA is used in experiments for modeling of AIDs such as uveitis and EAE [25]. The lipid components of CFA such as trehalose dimycolate (TDM, also known as cord factor) and mycolic-acid-containing glycolipids with strong adjuvant activity [85,86] have been shown to be a substantial factor of adjuvant activity [26]. TDM is a glycolipid in the mycobacterial cell envelope that was discovered in the 1950s as a most potent immune-stimulatory molecule [87].

Mycolic acids, important lipid components of the bacterial cell wall of Mycobacterium, have been demonstrated to be efficient adjuvant, and compared with CFA did not cause severe inflammatory responses induced by Th17. Instead of this, MA induced Th1-mediated moderate inflammation at the site of injection, activating dendritic cells by means of costimulatory molecules CD80/86 and CD40 and induction of promoting cytokines [88].

## 7. Mycobacterium Tuberculosis–Host Cell Interaction

Central to immune response is an interaction between host professional phagocytes and Mtb, which will determine development and outcome of TB. Alveolar Mphs are the host phagocytic cells that eliminate pathogens directly or indirectly, activating the host innate and adaptive immune responses without excessive inflammation and lung destruction [89].

Multiple receptors take part in endocytosis of Mtb: they are the complement receptor [90]; the monocyte-inducible C-type lectin (Mincle), identified as the receptor for TDM (trehalose-6,6′-dimycolate) [91]; surfactant protein A (Sp-A) and its receptors [92,93,94]; scavenger receptor [95]; mannose receptors [95,96]; and the DC-specific intercellular adhesion molecule-3-grabbing nonintegrin (DC-SIGN, CD209) [97,98]. DC-SIGN interactions with Mtb may be of benefit for either the pathogen or for the host due to restriction of tissue inflammation and immunopathology [99,100,101]. DC-SIGN is expressed on both wound-healing (IL-4-dependent) and regulatory (M-CSF-dependent) alternative (M2) macrophages [101]. Pattern-recognition receptors also respond to Mtb, among them the TLR-1, TLR-2, TLR-4, TLR-6, TLR7, and TLR-9 on Mphs and DCs, thereby driving phagocytosis, antigen presentation to T cells, and cytokine secretion [102,103,104].

Polymorphisms in TLRs affect human susceptibility to tuberculosis [27,28] and possibly to AI.

It was shown that the Mtb chaperone-like protein GroEL2 present on the Mtb cell envelope modulated Mph proinflammatory responses [105]. GroEL2 has been reported to be a major stimulator of immune response to Mtb-purified protein derivative (PPD) [106]. Cleavage of multimeric GroEL2 by the serine protease Hip1 resulted in the appearance of a cleaved form of GroEL2, which retarded innate immune responses to Mtb infection [107]. The full-length GroEL2 protein caused powerful proinflammatory responses activating DC maturation, antigen presentation to T cells, and inducing the Th1 subset development. The cleaved form of GroEL2 was unable to promote an efficient T-cell response [105]. The authors suggested that cleavage of GroEL2 averts optimal host response and that the prevalence of one of the two forms of GroEL2 during TB will determine the type of host immune response generated [105].

The microbial products can break self-tolerance and induce autoimmune manifestations, activating antigen-presenting cells. The development of EAE, even in genetically EAE-resistant mice, was observed after activation of APCs via TLR9 or TLR4 [108].

## 8. Unique Protein Family PE/PPE/PGRS Present on the Mtb Surface

Some molecules present on the Mtb surface are unique. The genome of Mtb encodes a protein family PE/PPE/PGRS, present exclusively in the genus Mycobacterium [109]. The complete genome sequence of the best-characterized strain of *Mycobacterium tuberculosis*, H37Rv, has been determined in 1998 by Cole et al. and a family of genes, the Proline–Glutamic acid/Proline–Proline–Glutamic acid (PE/PPE), has been identified [110]. These genes are principally characteristic of the pathogenic strains. The data on this important family of proteins are summarized in the review [29].

PE proteins are divided in three subfamilies: PE; PE/PPE; and PE_PGRS containing the polymorphic glycine-rich domain of variable sequence and size [29]. 

PE/PPE proteins have been reported to use the host inflammatory signaling and cell-death pathways to facilitate disease development [33]. It is widely recognized that PE_PGRS [polymorphic GC-rich-sequence (PGRS)] proteins are essential in TB pathogenesis [29,111,112]. 

The PE/PPE/PGRS are involved in cell-wall remodeling; they interfere with the host cell-death pathways and virulence [30], mycobacterial antigenic variation, immune evasion [31], and influence innate immunity and bacillary survival in macrophages [32,33]. Polymorphisms in the PE/PPE/PGRS protein family may influence different manifestations of TB, among them AI [112].

## 9. *Mycobacterium tuberculosis* Manipulates the Host Immune Response

The data showing that multiple molecules on the Mtb surface promote phagocytosis suggest that Mtb finds the intracellular environment of macrophages especially advantageous for surviving [113,114]. Mycobacteria manipulate host phagocytes to survive and replicate in these cells. PE_PGRS30 protein of Mtb blocks phagosome maturation [115]. Autophagy, a potent host defense mechanism, is impaired by several Mtb mechanisms [115,116,117,118,119]. PE_PGRS11 can induce maturation and activation of human DCs, which promotes the secretion of proinflammatory cytokines [120]. PE_PGRS17 binding to TLR2 activates the NF-κB signaling pathway, inducing TNF-α secretion [120]. 

Hyperactive immune response leads to robust inflammation, which induces dissemination and transmission of bacteria and possibly AI development.

## 10. PE_PGRS Proteins in TB Pathogenesis

Studies of pe_pgrs genes demonstrated that expression levels of different pe_pgrs genes could differ essentially [29], leading to a diverse picture and different outcome of TB. Each protein of the PE_PGRS family can fulfill its unique function without a specific protein partner. The identification of PE_PGRS proteins in Mtb and understanding their functions leads to the acknowledgement of their potent role in the TB pathogenesis [29]. It is possible to suggest the involvement of PE_PGRS proteins in AI promotion. 

## 11. Excessive Cell Death as a Possible Mechanism of Autoimmunity

Cell death is a substantial physiological and pathological process involved in coordination of immune responses and AI [34]. Normally after cells die they are quickly and smoothly removed by phagocytes without inflammation [121,122]. However, during chronic infection, a large number of cells die, releasing massive amounts of cellular contents into the extracellular space. Released molecules are known as danger-associated molecular patterns (DAMPs) acting as damage signals, which attract additional immune cells to clear the threat and promote tissue repair [34]. The latest discoveries in the pathways of cell death and their effects were summarized in [34,42]. 

Apoptosis is immunologically silent cell elimination without inducing inflammation due to containing the distracted contents of dying cells within membrane-bound vesicles called apoptotic bodies [45,121]. Many cellular signals can lead to cell death in a controlled manner [123]. The morphological changes during apoptosis are cytoskeletal disruption, cell shrinkage, DNA fragmentation, and plasma membrane blebbing [124]. Many nuclear autoantigens have been shown to accumulate within apoptotic blebs [125,126]. It was shown that apoptotic vesicles from Mtb-infected macrophages had potent adjuvant effects, stimulating CD8 T cells in vivo [127].

Apoptotic bodies are engulfed later by another phagocyte in a process termed efferocytosis [128,129]. ACs release “find me” signals such as soluble lysophosphatidylcholine, CXC3CL1, sphingosine-1-phosphate, ATP, and UTP that attract phagocytes for the clearance of apoptotic bodies [130]. It was shown that in TB, such a role plays CX3CL1 and its receptor CX3CR1 [131]. The best-studied signal “eat me” is an oxidized phosphatidylserine and oxidized low-density lipoprotein on the surface of the phagocyte [130,132]. Phosphatidylserine, a membrane component of ACs, plays an important role in the clearance of apoptotic bodies by the efferocytosis process [128,133]. 

Apoptosis of infected cells has been shown to stimulate self-reactive T cells promoting AI in infections [35], and TB is an example of an infection characterized by massive macrophage apoptosis serving as a potential principal mechanism.

Phagocytosis of infected apoptotic cells results in the presence within the same phagosome of both cellular and microbial antigens. This makes possible the presentation of self-antigens by major histocompatibility complex class II (MHC II) molecules, leading to the generation of autoreactive Th17 cells, associated with autoantibody production [35]. 

Bacterial infections also cause pyroptosis [38], programmed cell death, accompanied by osmotic lysis, followed by release of inflammatory cytokines and cell contents [37]. Both nuclear and mitochondrial DNA are released by pyroptotic cells [134]. Pyroptotic cells release an important inflammatory protein high-mobility group box 1 (HMGB1), a nuclear DNA-binding protein [39]. Complex HMGB1-DNA in vitro can stimulate TLR9 and type I IFN production by dendritic cells and activate B cells through the receptor for advanced glycation end-products (RAGE), facilitating autoreactive B-cell formation [40]. Cytokines caspase 1-dependent IL-1b and IL-18 released by pyroptosis are thought to promote AIDs [41]. 

## 12. Defective Dead Cell Clearance in Etiopathogenesis of Autoimmune Diseases

Infections have been shown to be linked with the onset of SLE [4,5]. The potential connection between infections and AI could be clearance deficiency [4]. Apoptotic cells are frequently not cleared adequately in SLE [46,135,136,137,138]; as a result, autoantigens are presented to B cells by follicular DCs in secondary lymphoid tissues [135,136,139]. Nucleic acids and the proteins binding to nucleic acids are the main autoantigens in the AID SLE [37]. Nuclear and membrane autoantigens accumulate in lymphoid organs and is thought to activate the autoreactive B and T cells, causing the production of antinuclear and antiphospholipoprotein AABs [139]. The production of antinuclear AABs and binding them to apoptotic nuclear remnants leads to chronic tissue damage, and development of systemic AIDs [136]. It was hypothesized that impaired phagocytosis in ANCA-associated vasculitis leads to accumulation of apoptotic neutrophils, which further are exposed to secondary necrosis, leading to AAB formation [47].

## 13. Modulation of Cell-Death Pathways by *Mycobacterium tuberculosis*

Among the various cell-death types in TB were documented apoptosis, pyroptosis, autophagy, and necrosis [42]. Impairment of apoptosis and autophagy provides a survival niche to Mtb [114,140]. Mycobacteria can modulate the death of the host cells. The popular opinion is that virulent Mtb inhibits apoptosis, while avirulent mycobacteria stimulate it. Virulent strains H37Rv and GC1237 are the most effective inhibitors of experimentally induced cell death. However opposite data from different experimental systems evidence that cell death results from complex interrelations of pro- and anticytotoxic mechanisms [141]. RipA, a secretory peptidoglycan hydrolase, damages both autophagy and apoptosis in Mph for intracellular survival and virulence [119].

Some of the PE/PPE/PGRS family proteins were reported to promote apoptosis of infected Mphs [44,45,109]; PE25–PPE41 complex and PE_PGRS33 induce necrosis and inflammation [142], tissue damage, and persistence in the lung tissue [112], resulting in dissemination of the disease [43,44]. On the other hand, M. tuberculosis genes nuoG and secA2 have been discovered to inhibit apoptosis [42]. 

Apoptosis is usually considered to be a protecting mechanism of the host against Mtb at the early stage of TB. During later stages, it may promote the disease dissemination in lung granulomas [109]. The PE_PGRS5 protein of Mtb presented exclusively in the pathogenic Mycobacterium genus has been demonstrated to induce the apoptosis of Mphs [109].

## 14. MerTK Is a Major Macrophage Apoptotic-Cell Receptor

There is a strict correlation between SLE disease severity and the activation of an M2-like macrophage expressing CD163 and MerTK during the monocyte-to-macrophage differentiation [36]. Mer tyrosine kinase (MerTK) has been reported to be a number one Mph apoptotic cell (AC) receptor. Its functional defect causes defective AC clearance promoting AI and atherosclerosis [143]. Mer tyrosine kinase (MerTK), a member of the TAM (Tyro3, Axl, Mer) subfamily of receptors, is specifically involved in removal of early ACs, recognizing unmodified phosphatidylserine. Deficiencies in TAM receptors may contribute to human autoimmune diseases [144].

MerTK is expressed in primary and secondary lymphoid organs and is responsible for both central and peripheral tolerance through multiple mechanisms: clearance of AC-derived potential autoantigens [145]; reduction of proinflammatory cytokines production [146]; prevention of autoreactive B- and T-cell expansion [147,148]. In SLE patients, diminished AC removal is believed to promote the production of AABs against apoptotic material [129,138]. These patients had reduced plasma levels of the MerTK ligand Protein S [149], which may explain functionally defective AC clearance [36].

Populations of phagocytes M2c (CD163+) Mphs remove ACs, including apoptotic immune cells in healthy individuals, and release anti-inflammatory cytokines [150]. M-CSF was found to differentiate Mphs in the presence of IL-10, which express high levels of MerTK; such Mphs have M2c phenotypes. Gene polymorphisms of MerTK and its ligand growth arrest-specific 6 (Gas6) are connected with clinical manifestations in SLE patients [151,152].

## 15. Macrophage Polarization Programs

Mature Mphs can undergo functional polarization in response to environmental signals. Two well-appreciated Mph polarization programs are (M1) induced by LPS+IFNγ, secreting IL-12 and promoting Th1 differentiation; (M2) Mphs that are induced by IL-4: (M2a), secreting IL-4 and inducing Th2 polarization; (M2b) and (M2c), both secreting IL-10 and linked with regulatory T-cell (Treg) propagation [153]. These cells can switch from one phenotype to another. They can either facilitate a proinflammatory or an anti-inflammatory effect, which makes them a potential participant in the development of AIDs [154]. 

M1 macrophages are known to have proinflammatory effects, and their cytokines mediate autoimmune and chronic inflammatory diseases. M2-like macrophages mainly have anti-inflammatory properties. However, recent studies also demonstrated pro-inflammatory functions of these cells. Both macrophage types take part in the pathogenesis of SLE (reviewed by [155]).

Mtb can activate infected Mphs and thus change the cytokines and chemokine production. The ESAT-6 (early Secreted Antigenic Target 6 kDa) is thought to be one of the Mtb factors inducing the proinflammatory M1 phenotype at the start of the infection, which facilitates granuloma formation and then switches M polarization from M1 to M2 at a later stage of the infection [156].

## 16. Immune Tolerance

Control of the T-cell tolerance to self-antigens carried out at several levels.

DCs present self-antigens to developing T cells in thymus and delete lymphocytes with autoreactivity [157]. Central tolerance control occurring in thymus through mechanism of selection leads to release into the circulation of high-affinity T cells specific for non-self-antigens, low-affinity T cells specific for self-antigens, and natural Treg (nTreg) with an intermediate affinity to both self- and non-self-antigens [158]. 

Two types of peripheral tolerance mechanism exist in a steady state after antigen capture by DCs [159]. One is the T-cell deletion involving activation of the programmed death 1 (PD-1) and the cytotoxic T-lymphocyte–associated antigen 4 (CTLA-4) on T cells [160]. Immune checkpoints CTLA-4 and PD-1 are negative regulators of T-cell immune function. A second is the induction of foxp3+ regulatory T cells (Tregs) [161,162,163]. Fms-like tyrosine kinase 3 ligand (flt-3L) is a hematopoietin necessary for expanding DC subsets and Tregs in vivo [164]. The use of flt-3L has been shown to be effective in treating AI in mice [164,165,166]. 

Dendritic cells—“specialized and regulated antigen processing machines” [167].

DCs in culture exist in two functionally and phenotypically distinct states: immature and mature. Immature DCs function as phagocytes and express relatively low levels of surface MHC class I and II and costimulatory molecules, and can not present antigen properly to T cells. Being activated by microbial products or proinflammatory cytokines, immature DCs transform into mature DCs, which are characterized by low phagocytic capacity but extreme capacity for T-cell stimulation [168].

## 17. Dendritic Cell Subsets

Several DC subsets have been identified by their ontogeny, phenotype, and transcriptional profile [169]. In humans, blood DCs are defined as CD303+, CD304+, CD123+, plasmacytoid DCs, and conventional DCs (cDCs), the latter divided into two subsets, the CD1c+ DCs and the CD141+ DCs [170]. More recently, a third subset of DCs, named monocyte-derived DCs (Mo-DCs), has been described in patients with RA and in other inflammatory states [171,172,173]. These cells differentiate from monocytes in inflamed tissues and induce Th1, Th17, or Th2 responses depending on the signal received [174]. 

All these subsets of DCs have been identified with altered phenotypes and functions in several chronic inflammatory/autoimmune disorders. Different changes of DC subsets were found in autoimmune disorders [175].

DCs are central regulators of the balance between immunity and tolerance, and alteration of the specialized DCs system is a common feature of both systemic and tissue-specific AIDs [170]. Plasmacytoid DCs in SLE patients produce high levels of INF-alpha, the specific cytokine of this disease, responsible for high activation of innate and adaptive immunity [176]. Pathogen signal molecules induce immunogenic DCs to promote effector functions of adaptive immune cells [177,178,179]. DC subsets and mechanisms involved in regulatory T-cell induction have been reviewed by [178].

The plasticity of DCs, dependent on different extents of maturity, may be used in cell-based therapy to restore immune tolerance in AIDs. The beneficial effect of tolerogenic DC (tolDC) has been demonstrated in autoimmune models in mice. They caused immune tolerance, resolution of immune responses and prevention of AI by inhibition of effector and autoreactive T cells and by promotion of Treg cells [179,180,181]. TolDCs have become promising cell-based therapies for treatment of AIDs [182,183,184,185,186].

## 18. Vitamin D, Autoimmunity, Tuberculosis

Vitamin D has been discovered to have an important immune-modulatory function, enhancing the innate and inhibiting the adaptive immune response and acting as an environmental factor facilitating AID development [52,53,54,55,56,57,60,187,188,189]. The optimal vitamin D concentration beneficial for health and preventing the risk of AIDs was declared to be 30–40 ng/mL 25(OH)D [190].

The vitamin D3 receptor (VDR) and the vitamin D3 activating enzyme 1-α-hydroxylase (CYP27B1) are expressed in many cell types, including immune cells, and thus they can produce active 1,25(OH)2D from circulating inactive 25(OH)D [191,192]. The 1,25(OH)2D then activates the VDR, which binds to nuclear receptors of the retinoic X receptor (RXR) family and induces antimicrobial peptides cathelicidin and defensins [193,194]. VDR gene polymorphisms influence susceptibility to pulmonary tuberculosis [195].

Vitamin D inhibits the maturation and antigen presentation of DCs [57,196] and changes the profile of T-helper cells (Th1, Th2, Th9, Th17) and Treg cells [197]. It was reported that vitamin D lowers Th1 cell function, leading to decreased production of TNF-alpha, IL-2, granulocyte macrophage colony-stimulating factor (GMCSF) and IFN-gamma [198,199]. However, vitamin D increases the differentiation and proliferation of Th2 and Treg cells, which in turn stimulates the production of their anti-inflammatory cytokines IL-4, IL-5, and IL-10, which further suppress the development of Th1, Th17, and Th9 cells, producing immune tolerance [200]. 

## 19. Influence of Vitamin D and Vitamin A on Dendritic Cells

It has long been known that metabolism of vitamin D and of vitamin A is an important regulator DC function [201]. VitD3 can cause DC tolerogenicity and suppress AIDs in murine models [202,203]. VitD3-induced CD141+ DCs had a stable CD83low immature phenotype even after exposure to an effective DC maturation cocktail consisting of TNF, IL-1β, IL-6, and PGE2 [204] and was characterized by poor T-cell stimulatory capacity [205]. The profitable effects of vitamin D3 treatment were received in EAE, an experimental model of MS [206].

The internal mechanism of the vitD3-induced immune-regulatory functions on DCs is the biological activity of IDO (Indoleamine 2,3-dioxygenase is a rate-limiting enzyme for the tryptophan catabolism). Both the injection of vitamin D3 and the adoptive transfer of vitamin D3-induced IDO + immature DCs result in a significant increase of amount of CD4+CD25+Foxp3+ regulatory T cells in the lymph nodes in a rat EAE [206]. Control of tryptophan metabolism by IDO in DCs is a regulator of innate and adaptive immune responses. In acute inflammatory reactions, cytokine IFN-γ induces IDO’s enzymatic function preventing harmful, exaggerated responses through the effects on tryptophan metabolism. IDO also can maintain the stable tolerance to self in a steady state, restraining AI [207,208]. 

Essential for the VitD3 reprogramming function is glucose oxidation and glycolysis activation [209,210], which is induced by recently identified as a critical checkpoint and direct transcriptional target of VitD3 glycolytic enzyme PFKFB4 (6-phosphofructo-2-kinase/fructose-2,6-biphosphatase 4) [209].

The use of vitamin D3 for the generation of tolDC was found as a most effective method among different others [209,210]. 

The important role played by retinoic acid has been evidenced in the in vitro generation and stabilization of Treg (Foxp3+RORγt+ T cell phenotype) as well as the immunosuppressive ability of such cells [211]. The cytokine transforming growth factor-beta (TGF-beta) converts naïve T cells into Treg cells that prevent AI, but in the presence of interleukin-6 (IL-6) TGF-beta stimulates the differentiation of naïve T lymphocytes into proinflammatory IL-17-producing Th17 cells, which induce AI and inflammation [212]. 

The vitamin A metabolite retinoic acid has been identified as a key regulator of TGF-beta-dependent immune responses, inhibiting the IL-6 induction of proinflammatory Th17 cells and stimulating anti-inflammatory Treg cell differentiation [211,212].

Immunosuppressive and anti-inflammatory agents are also known to promote tolerogenic DCs, which sometimes results in the expansion of regulatory T cells with suppressive activity. Low-molecular-weight drugs causing generation of tolDC could be used to better control different chronic inflammatory states such as AIDs or allograft rejection [213,214,215]. 

## 20. Effects of Vitamin D Analogs Supplementation in Autoimmune Diseases

The calcemic effect of calcitriol and VDR limits their clinical application; therefore, the invention of noncalcemic VDR ligands is needed to actualize the potential of VDR-targeting therapy [215]. Synthetic vitamin D analogs demonstrated protolerogenic potential, causing a significant reduction in IL-12p70 and IL23p19 as well as IL-6 and IL-17 production by the dendritic cells [216]. These data lead to the new approaches for treating inflammatory and AIDs. 

Nonsteroidal small-molecule compounds were discovered that activate the VDR, but are devoid of hypercalcemia [217].

## 21. Low Concentration of Vitamin D and Autoimmune Diseases

Vitamin D insufficiency is associated with AID development such as MS [52,53,57,60,187,188], RA [54,55,56,57,60,187,188], insulin-dependent diabetes mellitus [60,187], and IBD [53,57,58,59,60,187,218] (Table 2). The role of vitamin D in autoimmune diseases was reviewed by [57,60,187,188].

## 22. Tuberculosis, Vitamin D Deficiency, and Autoimmunity

High titers of various AABs present in pulmonary TB patients with vitamin D deficiency [48,49,50,51]. We revealed calcitriol deficiency and lack of proper cathelicidin response to infection in various forms of TB [48]. At the same time, these TB patients were characteristic of increased production of Th1 and Th17-derived cytokines and had blood prolactin level increased, which is well-known stimulator of AI [219]. These features taken together could be responsible for a greater inclination of TB patients to AI, and patients actually demonstrated increased levels of AABs towards several antigens, especially in more severe fibrous-cavernous forms of TB [48].

## 23. IL-17 and Related Cytokines in Inflammatory Autoimmune Diseases

Dysregulation of protective immune responses may cause AIDs. Excessive generation of Th17 cells resulting in high production of IL-17 may lead to AIDs [62,66,67]. IL-17 was found in many human AIDs, including MS, RA, SLE, IBD, and psoriasis [62,66,68]. Today, six homologous molecules are known (IL-17A–IL-17F). Activation of IL-17A and/or IL-17F induces the expression of IL-1, IL-6, IL-8, and TNF, and promotes the production of granulocyte colony-stimulating factor (G-CSF) and chemokines, that maintain chronic inflammation [63]. The IL-17 realizes the proinflammatory functions through the activation of NF-κB, MAPK, and C/EBP cascades [62].

## 24. Cytokine Promotion of Th-Cell Differentiation

Human Th-cell differentiation is largely regulated by IL-12, IL-23, and TGF-β. The CD4(+) T-cell subsets, Th1, Th2, Th9,Th17, Th22, and Treg cells are differentiated from naïve CD4(+) T cells depending on the cytokines they receive, and are characterized by the production of distinct cytokines [62]. Th1 cells, which are induced by IL-12 and IFN-γ, mediate host defense against intracellular pathogens by expressing IFN-γ. IL-6 plus TGF-β induces Th17 cells which express IL-17 and contribute to the eradication of extracellular bacteria [62,64,65].

Many data evidence that TGFβ and IL-6 are essential factors for the early stage of Th17 cell differentiation in mice [62,64], while IL-23 plays a central role in the functional maturation and maintenance of autopathologic Th17 cells [64,220]. IL-23 stimulates the differentiation and expansion of activated CD4+ cells that produce IL-17, IL-6, and TNFα upon antigen-specific stimulation. IL-23 is necessary for the generation of autoantigen-specific, highly pathogenic Th17 cells associated with AI [66,67,68]. IL-23 is also required for B cell follicle formation in the infected lungs and for long-term control of Mtb [220].

Other proinflammatory cytokines such as TNF-α and IL-1β together with Th17 cells/IL17 play significant roles in the pathogenesis of several autoimmune and chronic inflammatory diseases [62].

## 25. Th Cells and Cytokines in Tuberculosis

Th1 and Th17 are the main effector cells mediating protection and pathology during TB. Th1 cells have been established to facilitate protective action by secreting IFN-γ and activating Mphs. IFNγ has long been known as a regulator of T-cell responses in mycobacterial disease contributing to the elimination of mycobacteria-infected cells [65].

The function of Th17 cells during TB infection is complex because the pathogenesis of TB largely depends on the gravity of inflammation. Multiple data on Th17 actions in TB received both on mouse models and clinical TB show different results. Th17 induces chemokine and cytokine production, leading to neutrophil recruitment, tissue damage, and inflammation [65]. It was suggested that IL-17 may be protective during acute infection and detrimental during chronic ones [221] and in multidrug-resistant TB [63].

Heterogeneous cell populations Th1 and Th17 include subpopulations with diverse cytokine profiles playing different roles in immune pathology and protection. Th17.1 produces IFN-γ/TNF-α and IL-17 differentiating from Th17 in the presence of IL-12 and inflammatory cytokines, primarily IL-1β [65]. Th17.1 cells were found to be extremely pathogenic in the course of AIDs, but the role for these cells in active TB remains unclear. Th17.1 cells were detected in the broncho-alveolar fluid and lungs TB patients [65].

More recently, additional immune pathways were revealed, especially important is the role of type I interferons both in TB and in AIDs [16].

## 26. Conclusions

Multifactorial immune response against *Mycobacterium tuberculosis* includes immunologic, genetic, and environmental factors. Pathogenesis of TB and AI has many common immunological pathways that increase the chance to develop AI. More studies are needed to investigate these common pathways, and many questions remain unanswered. Comprehension of these mechanisms is necessary for the improvement of both TB and AID prognosis and treatment.

## Figures and Tables

**Table 1:** The autoantibodies in tuberculosis.

AAB Type	AAB in AIDs	AAB in TB (References)
rheumatoid factor (RF)	rheumatoid arthritis, Sjögren’s syndrome	[7,73,76]
antinuclear antibodies (ANA)	SLE, Sjögren’s syndrome, scleroderma, dermatomyositis	[7,8,50,70,72,73]
anti-dsDNA antibodies	SLE	[10,48,50,77]
antineutrophilic cytoplasmatic antibodies (ANCA)	ANCA-associated systemic vasculitis	[11,74,75]
anticyclic citrullinated peptide (anti-CCP)	rheumatoid arthritis	[76]
anti-Scl-70, antihistone antibodies	systemic sclerosis, SLE	[10]
antiphospholipid antibodies (aPL): the lupus anticoagulant (LA), anticardiolipin antibody (ACA), anti-beta 2 glycoprotein 1 (anti-ß2 GPI), anti-prothrombin	antiphospholipid syndrome, SLE	[21,22,24]
anticardiolipin antibody (ACA; IgM)	SLE, antiphospholipid syndrome	[8,10,11,50]
antibodies against β2 glycoprotein IgG	antiphospholipid syndrome, SLE	[11]
antibodies against proteinase 3, myeloperoxidase, bactericidal/permeability-increasing protein, lactoferrin	systemic vasculitis	[75]

AAB—autoantibodies, AID—autoimmune disease, TB—tuberculosis, SLE—systemic lupus erythematosus.

**Table 2:** Vitamin D deficiency and autoimmunity.

Association of Vitamin D deficiency with AIDs	References
MS	[52,53,57,60,187,188]
RA	[54,55,56,57,60,187,188]
Type 1 DM	[60,187]
IBDs	[53,57,58,59,60,187,218]
SLE	[60]
Thyrotoxicosis	[60]
Tuberculosis, vitamin D deficiency, and autoimmunity	[48,49,50,51]

MS—multiple sclerosis, RA—rheumatoid arthritis, DM—diabetes mellitus, IBD—inflammatory bowel disease, SLE—systemic lupus erythematosus.

## Data Availability

Not applicable.
