# Correction: Belyaeva et al. Tuberculosis and Autoimmunity. *Pathophysiology* 2022, *29*, 298–318

**DOI:** 10.3390/pathophysiology29030037

**Published:** 2022-08-16

**Authors:** Irina V. Belyaeva, Anna N. Kosova, Andrei G. Vasiliev

**Affiliations:** Department of Pathophysiology, St. Petersburg State Pediatric Medical University, Ministry of Healthcare of the Russian Federation, 194100 Saint Petersburg, Russia

The authors would like to make the following correction to the published paper [1]:

Correction of the reference order starting from reference 79 in the following sentence “Sjögren’s syndrome, SLE, RA, dermatomyositis, and polymyositis” at the end of heading 2, where reference 13 was replaced with reference 79, while reference 79 was replaced with reference 80. The reference order was corrected from this part until the end of the manuscript.

Besides, additional refences were added: reference 24 was added in the 1st sentence and reference 10 in the 2nd sentence of heading 4, 1st paragraph; reference 50 in the line of “antinuclear antibodies (ANA)” in Table 1 and references 70 and 73 in heading 2, 2nd paragraph, 1st sentence, after “antinuclear antibodies”; references 52–56 in the first sentence of the 1st paragraph, heading 18; reference 101 in heading 7, 2nd paragraph, after “alternative (M2) macrophages”; reference 158 in the end of 2nd paragraph, heading 16; reference 212 in the last sentences of the 5th paragraph, heading 19. Reference 211 in the 6th paragraphs in heading 19 should be changed to [211,212], reference 215 was added in the last sentence of the last paragraph, heading 19, so now there should be [213–215]. References 60,187,188 were added after “MS” and “RA” respectively, references 60 and 187 after “insulin-dependent diabetes mellitus” and “IBD” respectively in the paragraph of heading 21; reference 188 was added in lines of “MS” and “RA” in Table 2 in heading 21, and reference 188 in the last sentence of heading 21.

The name of the 1st author Tailleux, L. in reference 100 (doi:10.2174/1573395054065151) which was somehow lost in the list of references was added. The explanation “SLE—systemic lupus erythematosus” should be added in the footnotes of Tables 1 and 2 and after the 1st sentence, 14th paragraph of heading 1: “Infections can be connected with the onset of SLE”.

These references were additionally replaced: reference 11 with 73 in the line of “rheumatoid factor (RF)” in Table 1, reference 21 replaced with 75 in the last line of Table 1 and after “while anti-PR3 increased in most patients” in heading 4, 2nd paragraph. The abbreviation aCL in 1st and 2nd paragraphs of heading 4 should be replaced with ACA. The references cited in the line of “IBDs” in Table 2 should be updated to [53,57–60,187,218].

These references were removed: reference 11 in the 3rd line “anti-dsDNA antibodies” of Table 1; reference 51 after “insulin-dependent diabetes mellitus” in the paragraph of heading 21; reference 220 in the sentence “It was suggested that IL-17 may be protective during acute infection and detrimental during chronic ones” in the 2nd paragraph of heading 25. The 1st sentence of the 2nd paragraph in heading 2 “The prevalence of anti-CCP and IgM RF was found in sera of TB patients [50,75]” was removed.

The authors apologize for any inconvenience caused and state that the scientific conclusions are unaffected. This correction was approved by the Academic Editor. The original publication has also been updated.
